# Supplementary material for: Ligand Co‐Deposition in Focused Electron Beam Induced Nanoprinting: A Predictive Composition Model
Source: Small Methods. 2025 Dec 21;10(3):e01956. doi: 10.1002/smtd.202501956 (PMC12893292; doi:10.1002/smtd.202501956)
Supplement: Supplementary file 1 — Supporting Information [file SMTD-10-e01956-s001.docx]

Supporting Information

Ligand co-deposition in focused electron beam induced nanoprinting: a predictive composition model

Jakub Jurczyk^1,2,3*^, Leo Brockhuis^1^, Amalio Fernández-Pacheco^1^ and Ivo Utke^2*^

S1 Molecule and ligand surface coverages for different parameters

Table S1 shows the parameters used for calculations presented in graphs S1 and S2. They were chosen to represent a broad range of experimental parameters and thus different deposition regimes.

Table S1: Parameters used for calculation of figures S1 (coverages) and S2 (yields).

| Parameter | Molecule (ML) a) | Ligand (L) a) | Molecule (ML) b) | Ligand (L) b) | Molecule (ML) c) | Ligand (L) c) | Molecule (ML) d) | Ligand (L) d) |
| --- | --- | --- | --- | --- | --- | --- | --- | --- |
| Molecule flux *J* [#/(m^2^s] | $1.0\cdot{10}^{22}$ |  | $1.0\cdot{10}^{21}$ |  | $1.0\cdot{10}^{22}$ |  | $1.0\cdot{10}^{22}$ |  |
| Adsorption site density *N_0_* [m^-2^] | $2.0\cdot{10}^{18}$ | $2.0\cdot{10}^{18}$ | $2.0\cdot{10}^{18}$ | $2.0\cdot{10}^{18}$ | $2.0\cdot{10}^{18}$ | $2.0\cdot{10}^{18}$ | $2.0\cdot{10}^{18}$ | $2.0\cdot{10}^{18}$ |
| **Gas supply rate** $\boldsymbol{\nu}_{\boldsymbol{gas}}\boldsymbol{=}\boldsymbol{J}/{\boldsymbol{N}_{\boldsymbol{0}}}$ **[s^-1^]** | $5.0\cdot{10}^{3}$ |  | $5.0\cdot{10}^{2}$ |  | $5.0\cdot{10}^{3}$ |  | $5.0\cdot{10}^{3}$ |  |
| Electron flux *f* [#e/(m^2^s] | $1.5\cdot{10}^{24}$ |  | $1.5\cdot{10}^{24}$ |  | $1.5\cdot{10}^{24}$ |  | $5\cdot{10}^{22}$ |  |
| Cross section for dissociation *σ* [m^2^] | $1.0\cdot{10}^{-20}$ | $5.0\cdot{10}^{-20}$ | $2.0\cdot{10}^{-21}$ | $1.0\cdot{10}^{-20}$ | $1.0\cdot{10}^{-20}$ | $2.0\cdot{10}^{-21}$ | $5.0\cdot{10}^{-21}$ | $1.0\cdot{10}^{-20}$ |
| **Dissociation rate** $\boldsymbol{\nu}_{\boldsymbol{dis}}\boldsymbol{=\sigma f}$ **[s^-1^]** | $1.5\cdot{10}^{4}$ | $7.5\cdot{10}^{4}$ | $3.0\cdot{10}^{3}$ | $1.5\cdot{10}^{4}$ | $5.0\cdot{10}^{2}$ | $1.0\cdot{10}^{2}$ | $2.5\cdot{10}^{2}$ | $5.0\cdot{10}^{2}$ |
| Average desorption time *τ* [s] | $5.0\cdot{10}^{-4}$ | $8.0\cdot{10}^{-4}$ | $5.0\cdot{10}^{-5}$ | $5.0\cdot{10}^{-5}$ | $5.0\cdot{10}^{-4}$ | $4.0\cdot{10}^{-4}$ | $5.0\cdot{10}^{-4}$ | $4.0\cdot{10}^{-4}$ |
| **Desorption rate** $\boldsymbol{\nu}_{\boldsymbol{des}}\boldsymbol{=}\boldsymbol{1}/\boldsymbol{\tau}$ **[s^-1^]** | $2.0\cdot{10}^{3}$ | $1.25\cdot{10}^{3}$ | $2.0\cdot{10}^{4}$ | $2.0\cdot{10}^{4}$ | $2.0\cdot{10}^{3}$ | $2.5\cdot{10}^{2}$ | $2.0\cdot{10}^{3}$ | $2.5\cdot{10}^{3}$ |
| Parameters derived from analytical formulas | | | | | | | | |
| $\boldsymbol{\alpha}$ | $1.0$ |  | $1.0$ |  | $1.0$ |  | $1.0$ |  |
| $\boldsymbol{\theta}_{\boldsymbol{0}}$ | $7.1\cdot{10}^{-1}$ |  | $2.4\cdot{10}^{-2}$ |  | $7.1\cdot{10}^{-1}$ |  | $7.1\cdot{10}^{-1}$ | $0.0$ |
| $\boldsymbol{\theta}_{\boldsymbol{\infty}}$ | $2.2\cdot{10}^{-1}$ | $4.3\cdot{10}^{-2}$ | $2.1\cdot{10}^{-2}$ | $1.8\cdot{10}^{-3}$ | $3.4\cdot{10}^{-1}$ | $4.9\cdot{10}^{-1}$ | $6.5\cdot{10}^{-1}$ | $5.4\cdot{10}^{-2}$ |
| $\boldsymbol{\theta}_{\boldsymbol{\infty}}^{\boldsymbol{ML}}\boldsymbol{/}\boldsymbol{\theta}_{\boldsymbol{\infty}}^{\boldsymbol{L}}$ | $5.1$ |  | $11.7$ |  | $7.0\cdot{10}^{-1}$ |  | $12.0$ |  |
| $\boldsymbol{Y}_{\boldsymbol{max}}\boldsymbol{=\sigma\cdot}\boldsymbol{n}_{\boldsymbol{0}}$ | $2.0\cdot{10}^{-2}$ | $1.0\cdot{10}^{-1}$ | $4.0\cdot{10}^{-3}$ | $2.0\cdot{10}^{-2}$ | $2.0\cdot{10}^{-2}$ | $4.0\cdot{10}^{-3}$ | $1.0\cdot{10}^{-2}$ | $2.0\cdot{10}^{-2}$ |
| ${\boldsymbol{Y}_{\boldsymbol{max}}^{\boldsymbol{\Lambda}}\boldsymbol{/}\boldsymbol{Y}}_{\boldsymbol{max}}^{\boldsymbol{M}}$ | $5.0$ |  | $5.0$ |  | $2.0\cdot{10}^{-1}$ |  | $2.0$ |  |
| $\boldsymbol{Y}_{\boldsymbol{0}}\boldsymbol{=}\boldsymbol{Y}_{\boldsymbol{max}}\boldsymbol{\cdot}\boldsymbol{\theta}_{\boldsymbol{0}}$ | $1.4\cdot{10}^{-2}$ |  | $9.8\cdot{10}^{-5}$ |  | $1.4\cdot{10}^{-2}$ |  | $7.1\cdot{10}^{-3}$ | $0.0$ |
| $\boldsymbol{Y}_{\boldsymbol{\infty}}\boldsymbol{=}\boldsymbol{Y}_{\boldsymbol{max}}\boldsymbol{\cdot}\boldsymbol{\theta}_{\boldsymbol{\infty}}$ | $4.4\cdot{10}^{-3}$ | $4.3\cdot{10}^{-3}$ | $8.5\cdot{10}^{-5}$ | $3.6\cdot{10}^{-5}$ | $6.8\cdot{10}^{-3}$ | $2.0\cdot{10}^{-3}$ | $6.5\cdot{10}^{-3}$ | $1.1\cdot{10}^{-3}$ |
| $\boldsymbol{x}_{\boldsymbol{0}}^{\boldsymbol{M}}$ | $1.0$ |  | $1.0$ |  | $1.0$ |  | $1.0$ |  |
| $\boldsymbol{x}_{\boldsymbol{\infty}}^{\boldsymbol{M}}$ | $5.0\cdot{10}^{-1}$ |  | $7.0\cdot{10}^{-1}$ |  | $7.7\cdot{10}^{-1}$ |  | $8.6\cdot{10}^{-1}$ |  |
| Dwell time $\boldsymbol{t}_{\boldsymbol{i}}$ **[s]** | $4.5\cdot{10}^{-5}$ | $1.3\cdot{10}^{-5}$ | $4.2\cdot{10}^{-5}$ | $2.9\cdot{10}^{-5}$ | $1.3\cdot{10}^{-4}$ | $2.9\cdot{10}^{-3}$ | $1.4\cdot{10}^{-4}$ | $3.3\cdot{10}^{-4}$ |

Figure S1 shows the coverages of precursor molecules and detached ligands for different parameter sets. As can be noticed, the coverage of precursor molecules is always a decreasing function, the onset of which depends on the used deposition parameters, as mentioned in the main text. The pronounced peak in the coverage of detached ligands occurs for the set with the highest dissociation rate, see fig S1a. The highest ligand coverages take place for the lowest ligand desorption rate, see fig. S1c.


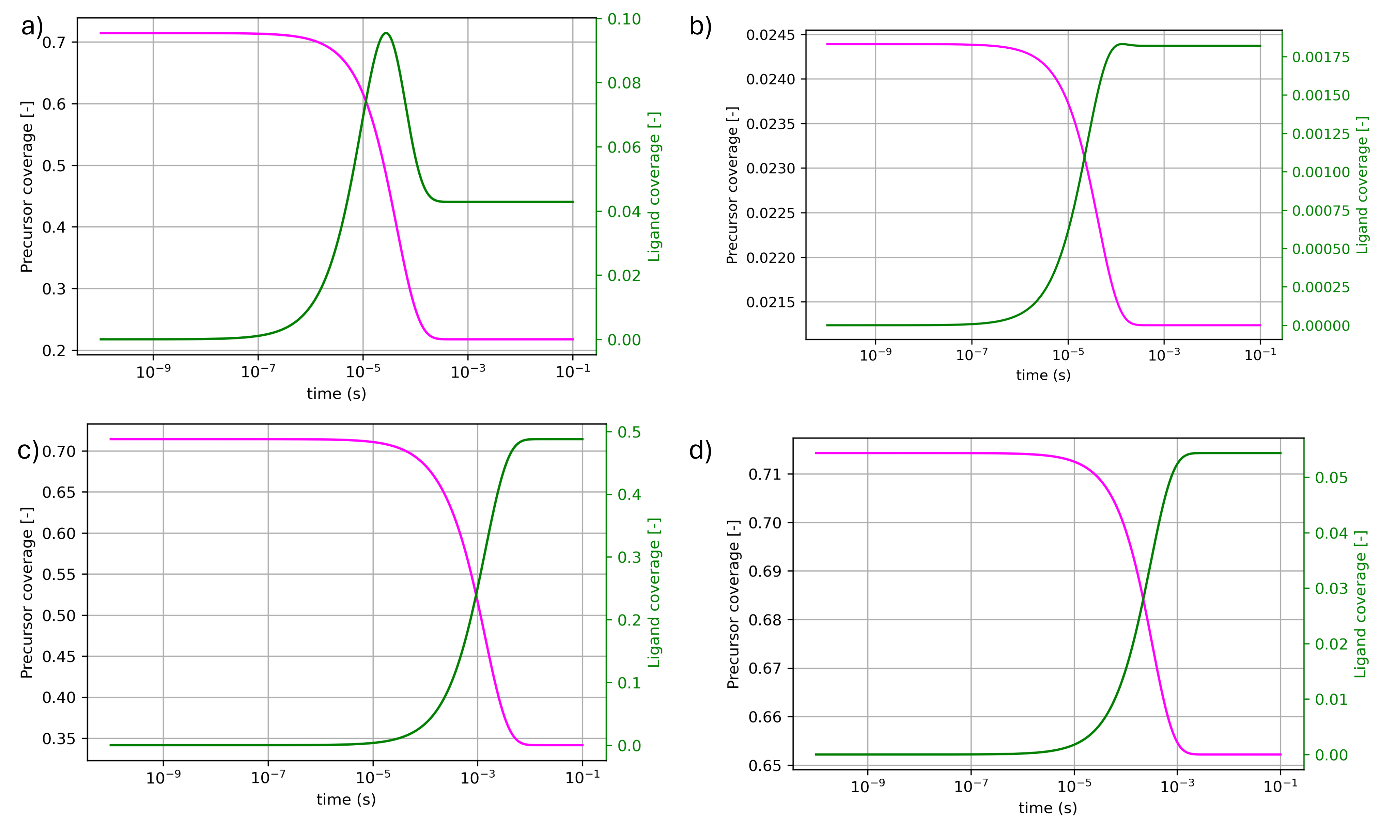


Figure S1. Precursor (purple) and detached ligand (green) coverages versus electron beam dwell time resulting from the parameter set of table S1.

S2 Yields and metal contents for different parameters

Figure S2 presents deposition yields and compositions calculated for the data set of table S1.

The presented data graphically confirms the observations i) to vi) stated in the main text at the end of section 4.2 for the yields and the statement of a monotonically decaying metal content with dwell time in the beginning of section 4.3. The lowest calculated steady state metal content is 50 at.% as $\mu=\lambda=1$ was set for the calculations in eqn. 37. The green region marks the most rapid changes in yields and composition. The upper horizontal axis with dwelling rate (which is defined as inverse of dwell time) was added to make it easier to position parameters like characteristic rates for different processes (gas supply, desorption, dissociation) on the scale.


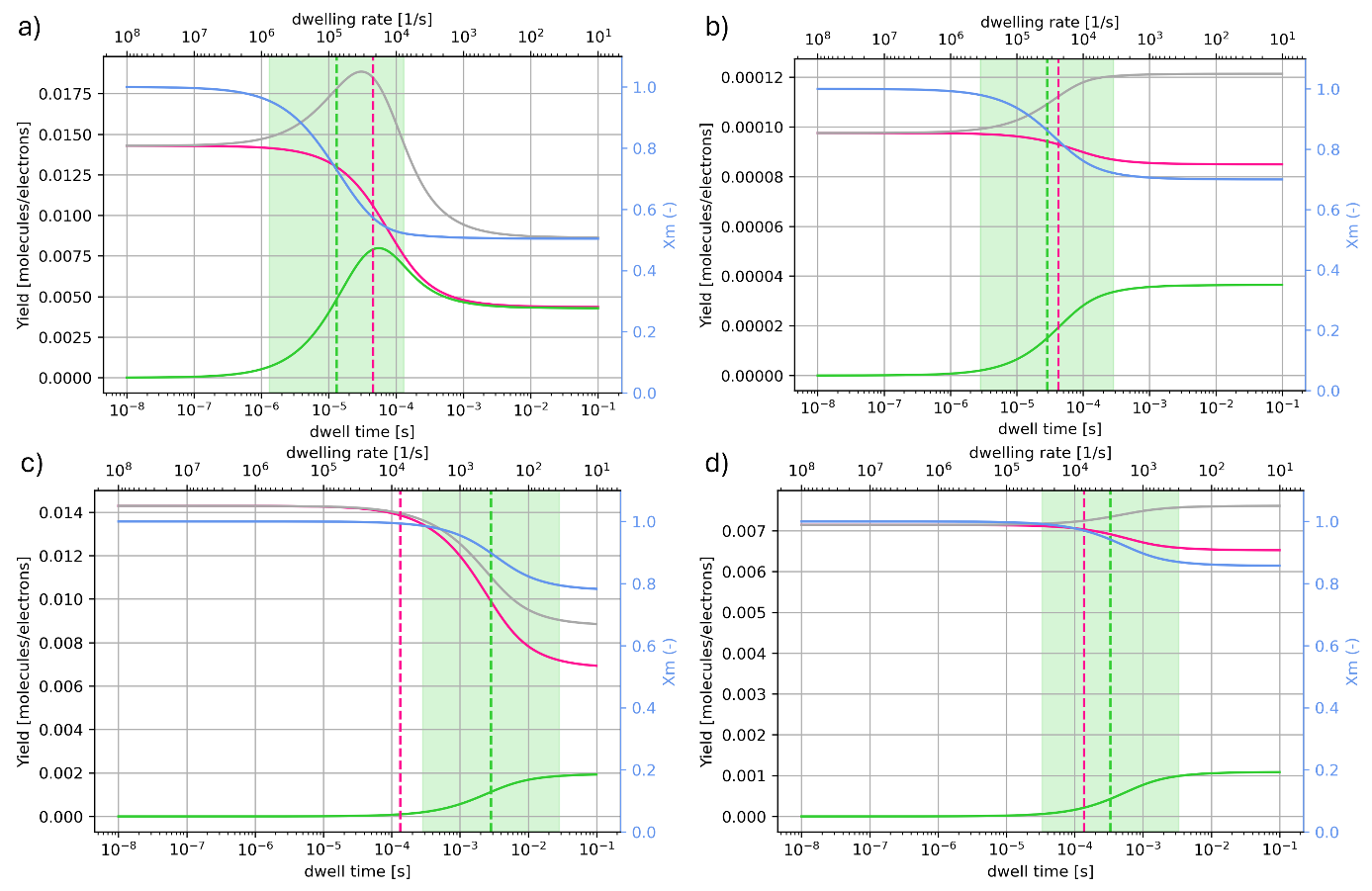


Figure S2. Calculated yields of metal (purple) and ligand (green), total yields (grey), and the metal content (light blue) in the nanoprinted material as function of electron beam dwell time. The parameter set of table S1 was used for calculations. The green vertical straight line stands for $1/\nu_{\Sigma}^{L}$ and the purple for $1/\nu_{\Sigma}^{ML}$. The green shaded area presents the range of $0.1t_{i}$ and $10t_{i}$.

S3 COMPARISON WITH THE STANDARD FEBID NANOPRINT MODEL

Since the solutions of the standard and ligand co-deposition models were derived from the same conceptual continuum rate approach, the total deposition yields for these two models should converge for the case where deposition of the detached ligand is minimal, i.e. forcing a very high $\frac{\nu_{des}^{L}}{\nu_{dis}^{L}}$ ratio. Hence, we use the well-established standard model, see eqns 2 to 9, as proof for the solutions derived for the yields in the ligand co-deposition model (eqns. 12 to 30).

The calculation parameters are summarized in table S2.

Table S2. Parameters for comparison with standard FEBID nanoprinting model. Note that the detached ligand's desorption and dissociation rates were chosen to result in very low deposition yield.

| Parameter | Molecule (ML) | Ligand (L) | |
| --- | --- | --- | --- |
| **Gas supply rate** $\boldsymbol{\nu}_{\boldsymbol{gas}}\boldsymbol{=}\boldsymbol{J}/{\boldsymbol{N}_{\boldsymbol{0}}}$ **[s^-1^]** | $5.0\cdot{10}^{2}$ | --- |  |
| **Dissociation rate** $\boldsymbol{\nu}_{\boldsymbol{dis}}\boldsymbol{=\sigma f}$ **[s^-1^]** | $3.0\cdot{10}^{3}$ | $1.5\cdot{10}^{3}$ | |
| **Desorption rate** $\boldsymbol{\nu}_{\boldsymbol{des}}\boldsymbol{=}\boldsymbol{1}/\boldsymbol{\tau}$ **[s^-1^]** | $1.5\cdot{10}^{3}$ | $2.0\cdot{10}^{5}$ | |
| Parameters derived from analytical formulas | | | |
| $\boldsymbol{\alpha}$ | $1.0$ | | |
| $\boldsymbol{\theta}_{\boldsymbol{0}}$ | $7.8\cdot{10}^{-1}$ | $0.0$ | |
| $\boldsymbol{\theta}_{\boldsymbol{\infty}}$ | $5.2\cdot{10}^{-1}$ | $7.8\cdot{10}^{-3}$ | |
| $\boldsymbol{\theta}_{\boldsymbol{\infty}}^{\boldsymbol{ML}}\boldsymbol{/}\boldsymbol{\theta}_{\boldsymbol{\infty}}^{\boldsymbol{L}}$ |  | $6.7\cdot{10}^{1}$ | |
| $\boldsymbol{Y}_{\boldsymbol{max}}\boldsymbol{=\sigma\cdot}\boldsymbol{n}_{\boldsymbol{0}}$ | $4.0\cdot{10}^{-3}$ | $2.0\cdot{10}^{-3}$ | |
| ${\boldsymbol{Y}_{\boldsymbol{max}}^{\boldsymbol{\Lambda}}\boldsymbol{/}\boldsymbol{Y}}_{\boldsymbol{max}}^{\boldsymbol{M}}$ |  | $5.0\cdot{10}^{-1}$ | |
| $\boldsymbol{Y}_{\boldsymbol{0}}\boldsymbol{=}\boldsymbol{Y}_{\boldsymbol{max}}\boldsymbol{\cdot}\boldsymbol{\theta}_{\boldsymbol{0}}$ | $3.1\cdot{10}^{-3}$ | $0.0$ | |
| $\boldsymbol{Y}_{\boldsymbol{\infty}}\boldsymbol{=}\boldsymbol{Y}_{\boldsymbol{max}}\boldsymbol{\cdot}\boldsymbol{\theta}_{\boldsymbol{\infty}}$ | $2.1\cdot{10}^{-3}$ | $1.6\cdot{10}^{-5}$ | |

Figure S3 presents such a comparison, where the deposition yield is calculated according to both the standard FEBID model and the ligand co-deposition model developed in this work. The dwelling rate axis was added to facilitate positioning of different characteristic rates on the scale.


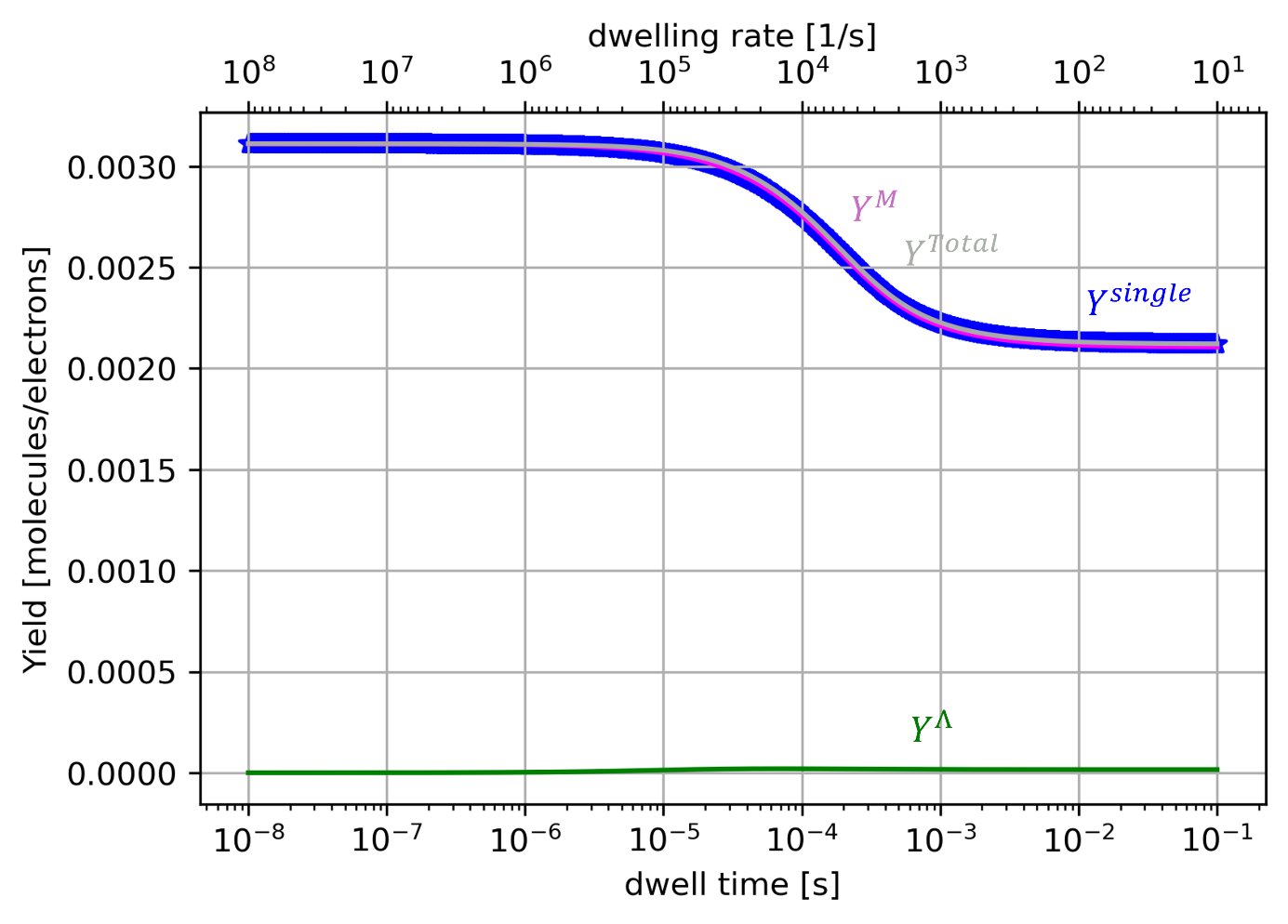


Figure S3. Comparison between deposition yield as a function of dwell time calculated using the standard single-species FEBID continuum model (dark blue stars) and the FEBID model developed in this work: metal yield (purple), ligand yield (green) and total yield (grey). The upper axis represents the dwelling rate.

The agreement between the total deposition yield of the ligand co-deposition model and the yield for the standard FEBID nanoprint model for the case of negligible ligand deposition yield proves the validity of solutions derived in eqns. 12 to 29. It further shows that the standard model (eqns. 2 to 9) can be treated as a special case of the ligand co-deposition model.

S4 PARAMETERS USED for modeling IN FIGURE 8

Parameters used to calculate yields and metal content versus electron beam dwell time for the hypothetical molecule presented in Figure 8 are summarized in Table S3.

**Table S3**: Parameters used for calculations in figure 8. Note that that ${\nu_{des}^{L}}/{\nu_{dis}^{L}}\approx5$ and $\nu_{des}^{L}+\nu_{dis}^{L}\approx4.75\cdot{10}^{4}$ were chosen to push the steady state metal content to high values and the onset of metal content decay to longer dwell times.

| Parameter | Molecule (ML) | Ligand (L) |
| --- | --- | --- |
| Molecule flux *J* [#/(m^2^s] | $1.0\cdot{10}^{22}$ |  |
| Adsorption site density *N_0_* [m^-2^] | $2.0\cdot{10}^{18}$ |  |
| **Gas supply rate** $\boldsymbol{\nu}_{\boldsymbol{gas}}\boldsymbol{=}\boldsymbol{J}/{\boldsymbol{N}_{\boldsymbol{0}}}$ **[s^-1^]** | $5.0\cdot{10}^{3}$ |  |
| Electron flux *f* [#e/(m^2^s] | $1.5\cdot{10}^{24}$ |  |
| Cross section for dissociation *σ* [m^2^] | $1.0\cdot{10}^{-21}$ | $5.0\cdot{10}^{-21}$ |
| **Dissociation rate** $\boldsymbol{\nu}_{\boldsymbol{dis}}\boldsymbol{=\sigma f}$ **[s^-1^]** | $1.5\cdot{10}^{3}$ | $7.5\cdot{10}^{3}$ |
| Average desorption time *τ* [s] | $5.0\cdot{10}^{-5}$ | $2.5\cdot{10}^{-5}$ |
| **Desorption rate** $\boldsymbol{\nu}_{\boldsymbol{des}}\boldsymbol{=}\boldsymbol{1}/\boldsymbol{\tau}$ **[s^-1^]** | $2.0\cdot{10}^{4}$ | $4.0\cdot{10}^{4}$ |
| Parameters derived from analytical formulas | | |
| $\boldsymbol{\alpha}$ | $1.0$ |  |
| $\boldsymbol{\theta}_{\boldsymbol{0}}$ | $2.0\cdot{10}^{-1}$ |  |
| $\boldsymbol{\theta}_{\boldsymbol{\infty}}$ | $1.9\cdot{10}^{-1}$ | $5.9\cdot{10}^{-3}$ |
| $\boldsymbol{\theta}_{\boldsymbol{\infty}}^{\boldsymbol{ML}}\boldsymbol{/}\boldsymbol{\theta}_{\boldsymbol{\infty}}^{\boldsymbol{L}}$ | $31.7$ |  |
| $\boldsymbol{Y}_{\boldsymbol{max}}\boldsymbol{=\sigma\cdot}\boldsymbol{n}_{\boldsymbol{0}}$ | $2.0\cdot{10}^{-3}$ | $1.0\cdot{10}^{-2}$ |
| ${\boldsymbol{Y}_{\boldsymbol{max}}^{\boldsymbol{\Lambda}}\boldsymbol{/}\boldsymbol{Y}}_{\boldsymbol{max}}^{\boldsymbol{M}}$ | $5.0$ |  |
| $\boldsymbol{Y}_{\boldsymbol{0}}\boldsymbol{=}\boldsymbol{Y}_{\boldsymbol{max}}\boldsymbol{\cdot}\boldsymbol{\theta}_{\boldsymbol{0}}$ | $4.0\cdot{10}^{-4}$ |  |
| $\boldsymbol{Y}_{\boldsymbol{\infty}}\boldsymbol{=}\boldsymbol{Y}_{\boldsymbol{max}}\boldsymbol{\cdot}\boldsymbol{\theta}_{\boldsymbol{\infty}}$ | $3.7\cdot{10}^{-4}$ | $5.9\cdot{10}^{-5}$ |
